# Female prediabetic rats are protected from vascular dysfunction: the role of nitroso and sulfide signaling

**DOI:** 10.1186/s40659-024-00575-1

**Published:** 2024-11-26

**Authors:** Sona Cacanyiova, Andrea Berenyiova, Hana Malinska, Martina Huttl, Irena Markova, Basak G. Aydemir, Veronika Garaiova, Martina Cebova

**Affiliations:** 1 Centre of Experimental Medicine, Institute of Normal and Pathological Physiology, Slovak Academy of Sciences, Bratislava, Slovakia; 2 Center for Experimental Medicine, Institute for Clinical and Experimental Medicine, Prague, Czech Republic

**Keywords:** Hereditary hypertriglyceridaemic rats, Sex, Perivascular adipose tissue, Hydrogen sulfide, Nitric oxide

## Abstract

**Background:**

The activity of perivascular adipose tissue (PVAT), a specific deposit of adipose tissue surrounding blood vessels, could contribute to sex differences in vascular tone control, particularly in dyslipidemic disorders; however, the mutual associations remain unclear. This study aimed to evaluate the relationships among sex, PVAT and vascular function in Wistar and hereditary hypertriglyceridemic (HTG) rats. Vasoactive responses of the isolated thoracic aorta with preserved or removed PVAT were compared in adult male and female Wistar and HTG rats, and the roles of nitric oxide (NO), hydrogen sulfide (H_2_S), cyclooxygenase (COX) and inflammatory signaling in vascular function were monitored in females.

**Results:**

HTG rats were hypertensive, but females less than males. Increased 2-h glycemia was observed in HTG rats regardless of sex; however, HTG females exhibited better glucose utilization than males did. Females, independent of strain, had better preserved endothelial function than males did. PVAT inhibited endothelium-dependent relaxation in all the rats except HTG females. In HTG males, pathologically increased aortic contractility was noted; however, in HTG females, the contractile responses were lower, thus approaching physiological levels despite the pro-contractile action of COX products. In HTG females, NO contributed to endothelial function to a lesser extent than it did in controls, but the presence of PVAT eliminated this difference, which corresponded with increased NO synthase activity. Although increased protein expression of several proinflammatory factors (TNFα, IL-6, iNOS, and NfκB) was confirmed in the aortic and PVAT tissue of HTG females, the protein expression of factors regulating the adhesion and infiltration of monocytes (ICAM-1 and MCP-1) was decreased in PVAT. Moreover, in HTG females, unlike in controls, H_2_S produced by PVAT did not inhibit endothelial relaxation, and regardless of PVAT, endogenous H_2_S had beneficial anticontractile effects, which were associated with increased protein expression of H_2_S-producing enzymes in both aortic and PVAT tissues.

**Conclusions:**

Despite increased inflammation and the pathological impact of cyclooxygenase signaling in female HTG rats, protective vasoactive mechanisms associated with milder hypertension and improved endothelial function and contractility linked to PVAT activity were triggered. Sulfide and nitroso signaling represent important compensatory vasoactive mechanisms against hypertriglyceridemia-associated metabolic disorders and may be promising therapeutic targets in prediabetic females.

## Introduction

Recent research has shown that premenopausal women under the age of 40 have a lower incidence of cardiovascular diseases associated with complications such as hypertension or diabetic vascular changes than men do [1]. Sex, reproductive state and genetic background seem to play important roles; however, the metabolic activity of perivascular adipose tissue (PVAT), a specific adipose tissue deposit surrounding blood vessel, may also contribute significantly to the observed differences due to its participation in vascular control. Under physiological conditions, PVAT has predominantly anticontractile effects that are induced by a transportable factor called adipocyte-derived relaxing factor, which could be hydrogen sulfide (H_2_S), an important gaseous transmitter [2]. The results obtained in recent years have shown that both PVAT and the sulfide signaling pathway can interfere with the etiopathogenesis of various cardiovascular and metabolic diseases. Obesity and diabetes could be associated with dysregulation of adipokine production, leading to the impaired anticontractile effect of PVAT. Beltowski [3] reported that in obese rats fed a high-calorie diet for 3 months, the anticontractile effect of PVAT was impaired, which was associated with reduced H_2_S production by PVAT. However, our previous results confirmed that, depending on the pathological conditions and degree of metabolic disorder, both PVAT and H_2_S can also act in different ways. In spontaneously hypertensive rats (SHRs), we confirmed the pro-contractile effect of H_2_S produced by the arterial wall, which, however, was balanced by compensatory stronger anticontractile activity triggered by PVAT [4]. Similarly, in hereditary hypertriglyceridemic (HTG) rats, a nonobese prediabetic model with genetically fixed hypertriglyceridemia, we demonstrated that H_2_S produced within the arterial wall contributed to endothelial dysfunction; however, the PVAT of HTG rats was associated with compensatory vasoactive mechanisms, which included stronger anticontractile action of H_2_S [5]. The balance between impaired regulation of vascular function and compensatory vasoactive mechanisms can also be affected by other factors, such as inflammation and/or female/male hormones. Souza-Paula et al. [6] reported that circulating H_2_S was increased in hypertensive pregnant rats, which was associated with stimulation of H_2_S generation in PVAT and anticontractile effects in the aorta. On the other hand, adipocyte

hypertrophy and increased secretion of proinflammatory cytokines from PVAT have been reported in a postmenopausal rat model [7]. Finally, PVAT is a complex tissue that, to maintain balance, can release contractile factors during vasorelaxation and, conversely, relaxing factors when contraction is initiated. Saxton et al. [8] showed in Wistar rats that PVAT had an anti-constrictor effect after stimulation of adrenergic receptors with noradrenaline, and conversely, a pro-constrictor effect on responses induced by acetylcholine. However, during pathological conditions, the balance can be shifted in different directions depending on the type of disorder and the current state.

Thus, PVAT can influence and regulate vascular function through the secretion of various substances, such as H_2_S, which directly affect vascular function. In contrast, vascular dysfunction can lead to increased secretion of inflammatory adipocytokines that induce insulin resistance, which is a risk factor for the development of endothelial dysfunction [9]. In male HTG rats, a disturbed redox balance combined with inflammation and altered nitric oxide (NO) bioavailability contributed to the observed endothelial dysfunction [10, 11]. Moreover, we previously confirmed that male HTG rats are protected by compensatory vasoactive mechanisms associated with PVAT activity and H_2_S production. However, data on the relationships between the sulfide signaling pathway, inflammation, and PVAT and its effect on vasoactivity in females are completely lacking. The aim of our work was to determine whether and how female sex affects selected metabolic and cardiovascular characteristics in HTG) rats, a strain characterized by an intermediate degree of metabolic disorder development (non-obesity, prediabetes) and mild hypertension. First, we compared blood pressure, biometric parameters, glucose utilization and the role of PVAT in the endothelial function and contractility of isolated thoracic aortas in normotensive Wistar and HTG male and female rats. Second, the roles of sulfide, nitroso, cyclooxygenase and inflammatory signaling pathways were determined by evaluating vasoactive responses; measuring the gene and protein expression of the respective enzymes; and assessing NO synthase activity in the aortic and PVAT tissues of female Wistar and HTG rats.

## Materials and methods

### Experimental animals and basic parameters

Eighteen- to twenty-week-old male and female Wistar rats (male: n = 8, female: n=8) and HTG rats (male: n=9, female: n=8) were used in this study. The body weight (BW) of each rat was determined before decapitation. Systolic blood pressure (BP) was measured in prewarmed rats via noninvasive plethysmography of the tail arteries before the beginning of the functional study. The animals were sacrificed by decapitation after brief anesthetization with CO_2_. To avoid variability in the level of sex hormones the female rats were sacrificed during the diestrus, the longest lasting phase of the cycle. After decapitation, aliquots of serum and tissue samples (heart, aortic arch, abdominal aorta, and perivascular fat) were removed, weighed, frozen in liquid nitrogen and stored at − 80 °C for biochemical analysis. The thoracic aorta (TA) was isolated for further functional examination in vitro.

The serum levels of lipids were measured via commercially available kits (Erba Lachema, Brno, Czech Republic; Roche Diagnostics, Mannheim, Germany). For the oral glucose tolerance test (OGTT), blood glucose was determined after intragastric administration of a glucose load (300 mg/100 g b.wt.) following overnight fasting. Blood was drawn from the tail before the glucose load at 0 min and thereafter at 30, 60, and 120 min.

### Functional study

The vessels were divided into two groups, vessels without PVAT (A–) and vessels with intact PVAT (A+), to distinguish between the contribution of H_2_S produced by PVAT and the effect of total H_2_S produced by the arterial wall and surrounding perivascular fat. The TA (descending part of the TA beginning below the aortic arch was subsequently isolated, cleaned of connective tissue and cut into 5 mm long rings. In rings (A–), the perivascular fat was removed from the arterial surface with fine scissors under a microscope, with caution not to damage the adventitia. In rings (A+), a continuous layer of perivascular fat (1 to 1.2 mm in width) was left around the vessel. The TA rings were vertically fixed between two stainless steel wire triangles and immersed in a 20 mL incubation organ bath with oxygenated (95% O_2_; 5% CO_2_) Krebs solution (118 mmoL/L NaCl; 5 mmoL/L KCl; 25 mmoL/L NaHCO_3_; 1.2 mmoL/L MgSO_4_.7H_2_O; 1.2 mmoL/L KH_2_PO_4_; 2.5 mmoL/L CaCl_2_; 11 mmoL/L glucose; 0.032 mmoL/L CaNa_2_EDTA) and kept at 37 °C. The upper wire triangles affixed to the TA ring were connected to isometric tension sensors (FSG-01, MDE, Budapest, Hungary), and changes in tension were registered by an NI USB-6221 AD converter (National Instruments, Austin, TX, USA and MDE, Budapest, Hungary) and S.P.E.L. Advanced Kymograph software (MDE, Budapest, Hungary). A resting tension of 1 g was applied to each ring and maintained throughout a 45- to 60-min equilibration period. Single concentrations of noradrenaline (NA; 10^–6^ mol/L) and acetylcholine (Ach; 10^–5^ mol/L) were added to the organ bath to test the integrity of the arterial wall (the contractile ability and integrity of the endothelium). After washing with physiological Krebs solution and an equilibration period, experiments with NA were started to obtain contractile responses. Adrenergic contractions were determined in the TA as the responses to cumulatively applied exogenous NA (10^–10^–10^–5^ mol/L). The contractile responses were expressed as the active wall tension in grams and normalized to the length of the respective ring preparation (mm). To examine endothelium-dependent vasorelaxation, increasing concentrations of acetylcholine (Ach; 10^–10^–10^–5^ mol/L) were applied in a cumulative manner to NA- precontracted aortic rings. The rate of relaxation was expressed as a percentage of the maximum NA- induced contraction.

Next, we examined the participation of certain signaling pathways in the vasoactive responses of the TA. To investigate the participation of COX products in the vasoactive responses, the COX inhibitor indomethacin (INDO) was used (10^–5^ mol/L). To determine the role of the endogenous NO pathway, the rings of the TA were incubated with a nonspecific inhibitor of NO synthase, N^G^-nitro-L-arginine methyl ester (L-NAME, 10^–5^ mol/L). The H_2_S scavenger bismuth(III) subsalicylate (BSC, 10^–5^ mol/L) was used to evaluate the participation of H_2_S in the vasoactive responses. To determine the effects of the inhibitors on contractile responses and endothelium-derived relaxation, all the mentioned compounds were acutely incubated for 20 min in an organ bath and the concentration–response curves to NA (10^–10^–10^–5^ mol/L) and Ach (10^–10^–10^–5^ mol/L) were repeated.

### Total NO synthase activity

Total NOS activity was determined in crude homogenates of the aorta and PVAT by measuring the formation of [^3^H]-L-citrulline from [^3^H]-L-arginine (ARC, St. Louis, MO, USA) as previously described and slightly modified by Pechanova et al. [12]. [^3^H]-L-Citrulline was measured with the Quanta Smart TriCarb Liquid Scintillation Analyzer (Packard Instrument Company, Meriden, CT). NOS activity was then normalized to protein content and expressed as picokatal per gram of protein (pkat/g protein).

### RNA isolation and determination of gene expression

Total RNA was isolated from the aorta and PVAT using RNA Blue (Top-Bio, Czech Republic). Reverse transcription and quantitative real-time PCR analysis were performed using the TaqMan RNA-to-CT 1- Step Kit, the TaqMan Gene Expression Assay (Applied Biosystems, USA), and the ViiA 7 Real-Time PCR System (Applied Biosystems, USA). The following TaqMan probes were used: Nos3 Rn02132634_s1, Cse Rn00567128_m1, Mcp-1 Rn00580555_m1, Tnfα Rn99999017_m1, NfκB Rn01399572_m1. Relative expression was determined after normalization against Hprt Rn01527840_m1 as the internal reference and calculated via the 2^–ΔΔCt^ method. The results were run in triplicate.

### Western blotting

PVAT and aortic samples were homogenized on ice in 0.05 M Tris buffer (pH 7.4) supplemented with protease inhibitors. The protein concentrations were determined via a Lowry assay. Proteins (20 µg total protein) were separated by 12% or 15% SDS‒PAGE depending on the size of the protein being measured and transferred to nitrocellulose membranes. The membranes were blocked with 5% milk in Tris- buffered saline containing Tween 20 (TBS-T). Afterward, the membranes were incubated with a rabbit polyclonal anti-eNOS antibody (Abcam, Cambridge, UK; dilution 1:1000), a rabbit polyclonal anti-iNOS antibody (Proteintech^®^, Manchester, UK; dilution 1:1000), a rabbit polyclonal anti-CBS antibody (Proteintech^®^, Manchester, UK; dilution 1:3000), a mouse monoclonal anti-CSE antibody (Proteintech^®^, Manchester, UK; dilution 1:5000), a mouse monoclonal anti-TNFα antibody (Proteintech^®^, Manchester, UK; dilution 1:3000), a mouse monoclonal anti-NFκB antibody (Cell Signaling, Danvers, MA, USA; dilution 1:1000), a rabbit polyclonal anti-Mcp-1 antibody (Abcam, Cambridge, UK; dilution 1:1000), a rabbit polyclonal anti-ICAM antibody (Proteintech^®^, Manchester, UK; dilution 1:1000), or a rabbit polyclonal anti-COX2 antibody (Proteintech^®^, Manchester, UK; dilution 1:500) overnight at 4 °C. All the blots were reprobed with a rabbit polyclonal anti-β-actin antibody (Abcam, Cambridge, UK; dilution 1:5000) overnight at 4 °C. The membranes were incubated with anti-rabbit (Abcam, Cambridge, UK) or anti-mouse (Cell Signaling, Danvers, MA, USA) secondary peroxidase-conjugated antibodies at room temperature for 2 h. Both the primary and secondary antibodies were diluted in TBS-T containing 1% milk. The signals were visualized with Clarity Western ECL Substrate (Bio-Rad, Inc., Hercules, CA, USA) via a ChemiDocTM Touch Imaging System (Bio-Rad) and quantified with Image Lab Software. The target protein amounts were normalized to those of β-actin and are presented in arbitrary units (a.u.).

### Statistical analysis

The group size was calculated via a priori analysis via G*Power software v3.1 [13], and the total sample size was calculated to 32 (n = 8/group). The normality of the data was tested via the Shapiro‒ Wilk test. Three-way analysis of variance (ANOVA) for repeated measurements with the Bonferroni post hoc correction was used to evaluate the vasoactive responses. Two-way ANOVA was used to evaluate the cardiovascular and biochemical data. One-way ANOVA was used to evaluate the NO synthase activity and gene and protein expression data. Data are expressed as the means ± S.E.M.s and were analyzed via OriginPro (OriginLab Corporation, Northampton, MA, USA). Differences between means were considered significant at P < 0.05.

### Drugs

The following drugs were used: acetylcholine, bismuth(III) subsalicylate, indomethacin, and N^G^- nitro-L-arginine methyl ester, all from Merck (Darmstadt, Germany), and noradrenaline from Zentiva (Prague, Czech Republic). Acetylcholine, noradrenaline and N^G^-nitro-L-arginine methyl ester were dissolved in distilled water. Bismuth(III) subsalicylate was dissolved in dimethyl sulfoxide. Indomethacin was first solubilized in 0.2 mol/L Na_2_CO_3_ and then dissolved in distilled water.

## Results

### Blood pressure, biometric parameters and lipid profile

**Fig. 1:**
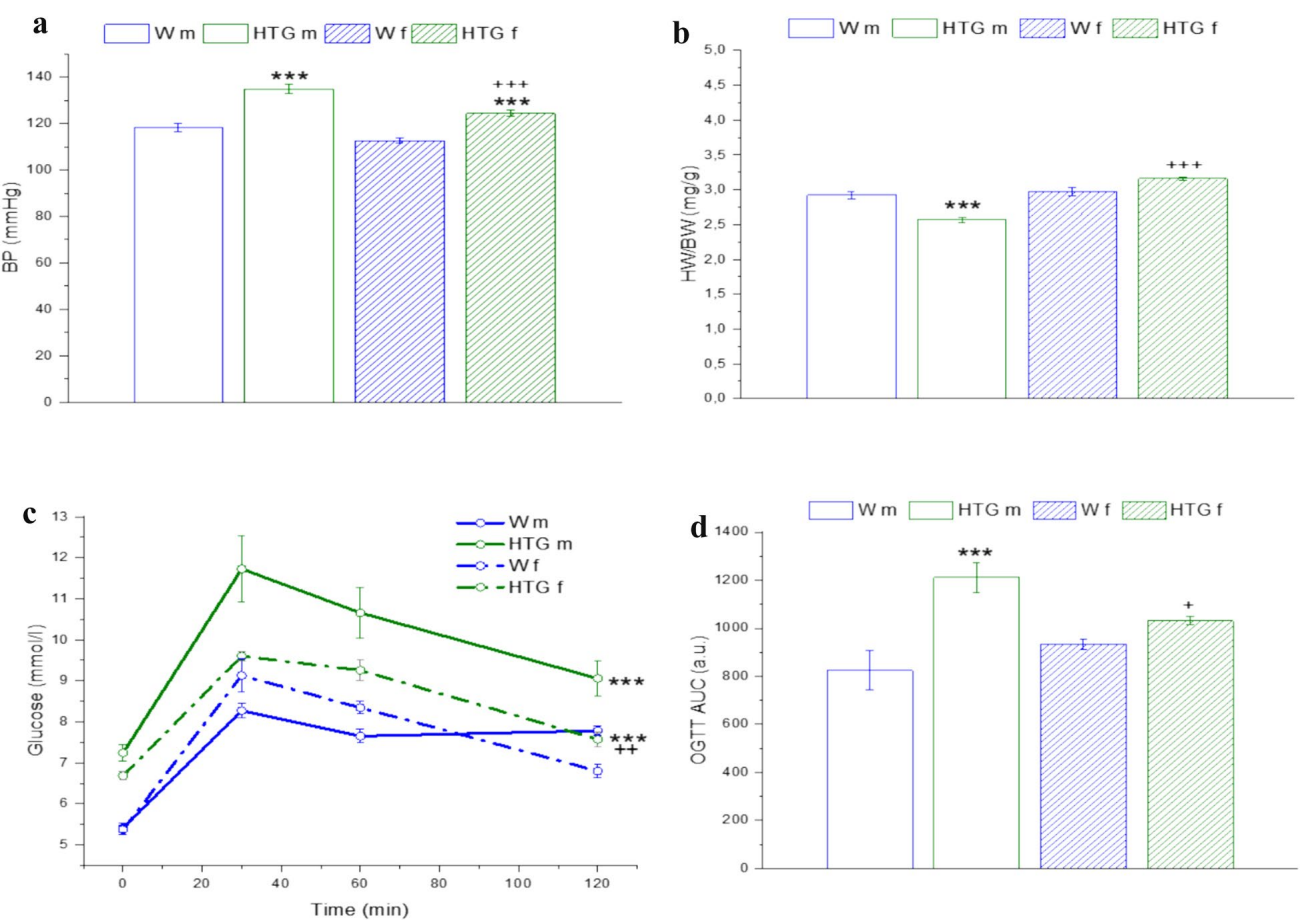
Systolic blood pressure (**a**), relative heart weight (**b**) and glucose utilization (**c**, **d**) in Wistar and hereditary hypertriglyceridemic rats. *Wm* Wistar male rats, *HTGm* hereditary hypertriglyceridemic male rats, *Wf* Wistar female rats, *HTGf* hereditary hypertriglyceridemic female rats, *BP* systolic blood pressure, *HW/BW* ratio of heart weight to body weight, *OGTT AUC* area under curve for glucose from oral glucose tolerance test. The results are presented as the mean ± S.E.M., and differences between groups were analyzed by two-way (**a**, **b, d**) or three-way (**c**) ANOVA. ***p < 0.001 vs. W within the respective sex, ^+^p < 0.05, ^++^p < 0.01, ^+++^ p<0.001vs. HTGm

The systolic blood pressure (BP) values were significantly greater in both HTG male (p < 0.001) and HTG female (p < 0.001) rats than in control Wistar rats; moreover, hypertriglyceridemic females had significantly lower BP values than males did (p < 0.001) (Fig. 1a). Two-way ANOVA revealed a significant effect of dyslipidemia (F_(1,230)_ = 63.32; p = 8.19×10^–14^) and sex (F_(1,230)_ = 20.25; p = 1.08×10^–5^) on BP values. Whereas the body weights (BWs) of male Wistar and HTG rats were comparable, the BWs were significantly lower in female HTG rats than in Wistar rats (p < 0.01); at the same time, all females had significantly lower BWs than males regardless of dyslipidemia status (both p < 0.001). Two-way ANOVA revealed a significant effect of dyslipidemia and sex on the BW values (Table 1). Heart weight (HW) was significantly lower in male HTG rats (p < 0.001) than in control Wistar rats and in both Wistar (p < 0.001) and HTG (p < 0.001) female rats than in males, regardless of dyslipidemia status. Two-way ANOVA revealed a significant effect of dyslipidemia and sex on HW values (Table 1). On the other hand, whereas the ratio of HW to BW was significantly lower in male HTG rats than in male Wistar rats (p < 0.001) and in female HTG rats (p < 0.001), confirming myocardial hypotrophy, the HW to BW ratio was comparable in female HTG and Wistar rats (Fig. 1b). With respect to myocardial remodeling, 2-way ANOVA revealed a significant effect of sex (F_(1,31)_ = 48.24; p = 1.24×10^–7^) only. Although the Bonfferoni post hoc test revealed no differences in the total cholesterol (Chol) values among the groups, 2-way ANOVA confirmed a significant effect of dyslipidemia (Table 1). However, the levels of HDL cholesterol (HDL-C) were significantly lower in hypertriglyceridemic rats than in control rats regardless of sex (males: p < 0.001; females: p < 0.001), and 2-way ANOVA confirmed the effects of both dyslipidemia and sex in this regard (Table 1). On the other hand, the levels of triacylglycerols (TAGs) were significantly greater in the hypertriglyceridemic rats than in the control rats, regardless of sex (males: p < 0.001; females: p < 0.001), and 2-way ANOVA confirmed the effect of dyslipidemia but not sex in this regard (Table 1).

**Table 1:** Biometrical and lipid parameters in Wistar and HTG rats

	Bonfferoni post hoc test				Two-way ANOVA	
Parameters	Wm	HTGm	Wf	HTGf	Sex	Strain
n	8	9	8	8		
BW (g)	424.5±10.77	413.7±8.69	289.5±3.88^+++^	250.5±3.05**^+++^	F _(1, 31)_ = 398.89; p = 1.7 × 10^–18^	F _(1, 31)_ = 11.14; p = 0.002
HW (g)	1.24±0.03	1.06±0.02***	0.86±0.02^+++^	0.79±0.01^+++^	F _(1, 31)_ = 233.47; p = 2.08 × 10 ^−5^	F _(1, 31)_ = 34.02; p = 2.53 × 10^–6^
Chol (mmol/L)	1.74±0.11	1.61±0.06	2.03±0.13	1.67±0.10	F _(1, 33)_ = 3.09; p = 0.09	F _(1, 33)_ = 6.44; p = 0.02
HDL-C (mmol/L)	1.27±0.08	0.87±0.02***	1.17±0.08	0.69±0.04***	F _(1, 30)_ = 4.99; p = 0.03	F _(1, 30)_ = 50.46; p = 9.96 × 10^–8^
TAG (mmol/L)	1.57±0.12	5.26±0.42***	1.58±0.04	5.90±0.16***	F _(1, 39)_ = 176.54; p = 0.29	F _(1, 39)_ = 1.13; p = 1.18 × 10^–15^

The results are presented as the mean ± S.E.M., and differences between groups were analyzed by two-way ANOVA. ** p < 0.01, ***p < 0.001 vs. W within the respective sex, ^+++^p < 0.01 vs. m within the respective strain

*BW* body weight, *HW* heart weight, *HW/BW* ratio of heart weight to body weight, *OGTT AUC* the area under the glycemic curve during the oral glucose tolerance test, *Chol* total cholesterol, *HDL-C* high-density lipoprotein cholesterol, *TAG* triacylglycerols, *n* number of rats, *Wm* Wistar male rats, *HTGm* hereditary hypertriglyceridemic male rats, *Wf* Wistar female rats, *HTGf* hereditary hypertriglyceridemic female rats

### Oral glucose tolerance test

To determine glucose utilization, we performed an oral glucose tolerance test (OGTT) (Fig. 1). Three-way ANOVA revealed a significant effect of dyslipidemia (F_(1,110)_ = 62.14; p = 5.03×10^–12^) and sex (F_(1,110)_ = 16.44; p = 1.02×10^–4^) on glucose utilization. Subsequent post hoc analysis revealed that while no difference was observed in Wistar rats, HTG males had significantly greater glycemia than HTG females did (p < 0.001, Fig. 1c). A similar marked tendency for 2-h glycemia to increase was observed in hypertriglyceridemic rats compared with Wistar rats, regardless of sex (males: p < 0.001, females: p < 0.01; Fig. 1c). The calculated AUC for glucose from the OGTT data was significantly greater in male HTG rats than in male Wistar (p < 0.001) and female HTG (p < 0.05) rats, and 2-way ANOVA confirmed a significant effect of dyslipidemia (Fig. 1d).

### The role of sex and PVAT in endothelial function and contractility

**Fig. 2:**
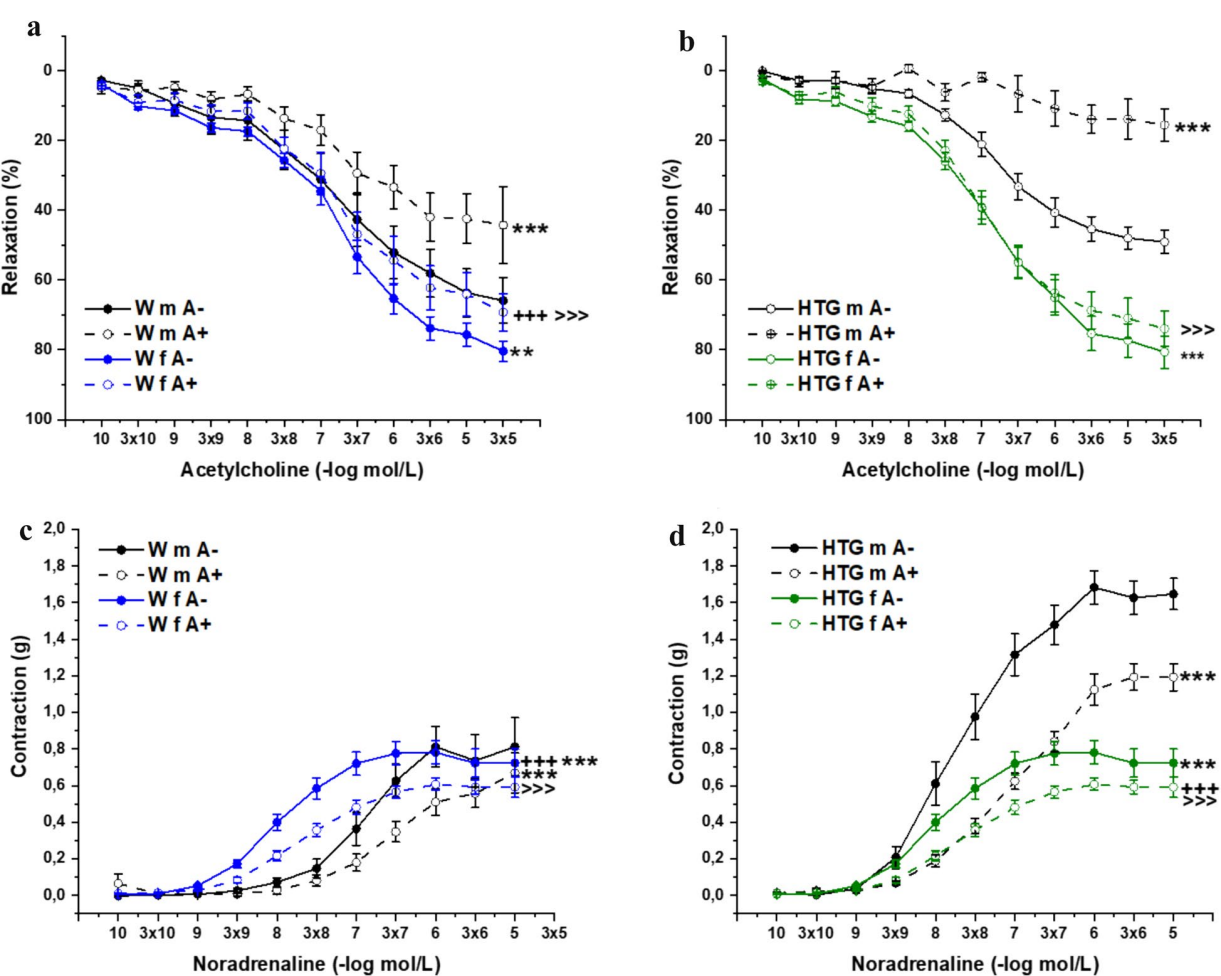
The role of sex and PVAT in endothelium-dependent vasorelaxation (**a**, **b**) and adrenergic contraction (**c**, **d**) of the isolated thoracic aorta in Wistar and HTG rats. *Wm* Wistar male rats, *HTGm* ereditary hypertriglyceridemic male rats, *Wf* Wistar female rats, *HTGf* hereditary hypertriglyceridemic female rats. A- perivascular adipose tissue denuded rings, A + -perivascular adipose tissue preserved rings. The results are presented as the mean ±S.E.M., and differences between groups were analyzed by three-way ANOVA. **p<0.01, ***p < 0.001 vs m A- within the respective strain, ^+++^p < 0.001 vs f A- within the respective strain, ^>>>^p < 0.001 vs m A+ within the respective strain

An evaluation of endothelial function revealed that the application of Ach (10^−10^–10^−5^ mol/L) relaxed the NA-precontracted TA rings of both strains and sexes. In Wistar rats, three-way ANOVA revealed a significant effect of sex (F_(1,497)_ = 36.07; *p* = 3.91×10^–9^) and PVAT (F_(1,497)_ = 28.46; *p* = 1.52×10^–7^) on the vasorelaxant response to Ach. PVAT significantly inhibited the vasorelaxant response in both male (*p*< 0.001) and female (*p*< 0.001) rats. Additionally, regardless of the presence of PVAT, Ach-induced

relaxation was significantly lower (*p*< 0.01 for A- groups; p< 0.001 for A+ groups) in male rats than in female rats (Fig. 2a). Similarly, in HTG rats, three-way ANOVA revealed a significant effect of sex (F_(1,550)_ = 385.19; *p* → 0) and PVAT (F_(1,550)_ = 59.73; *p* = 5.94×10^–14^) on the vasorelaxant response to Ach. However, regardless of the presence of PVAT, Ach-induced relaxation was significantly lower (*p*< 0.001 for A- groups; p< 0.001 for A+ groups) in male rats than in female rats, whereas PVAT significantly inhibited the vasorelaxant response in males only (*p*< 0.001) rats only (Fig. 2b).

With respect to contractility, the cumulative application of exogenous NA (10^−10^–10^−5^ mol/L) induced vasoconstriction in a concentration-dependent manner. In Wistar rats, three-way ANOVA revealed a significant effect of sex (F_(1,414)_ = 63.74; *p* = 1.75×10^–14^) and PVAT (F_(1,414)_ = 91.52; *p* → 0) on the contractile response to NA. PVAT significantly inhibited the contractile response in both male (*p*< 0.001) and female (*p*< 0.001) rats. Additionally, regardless of the presence of PVAT, NA-induced contraction was significantly lower (*p*< 0.001 for A– groups; p< 0.001 for A+ groups) in male rats than in female rats, although the maximum responses remained unchanged and only the dose‒response curves were shifted to the right (Fig. 2c). Three-way ANOVA also revealed a significant effect of sex (F_(1,484)_ = 260.09; *p* → 0) and PVAT (F_(1,484)_ = 212.0; *p* → 0) on the contractile responses of HTG rats to NA. PVAT significantly inhibited the contractile responses in both male (*p*< 0.001) and female (*p*< 0.001) rats. However, unlike in Wistar rats, NA-induced contraction was significantly lower (*p*< 0.001 for A- groups; p< 0.001 for A+ groups) in female rats than in male rats regardless of the presence of PVAT, which was related mainly to the different maximum responses achieved (Fig. 2d).

### The role of dyslipidemia and PVAT in the endothelial function and contractility of female Wistar and HTG rats

**Fig. 3:**
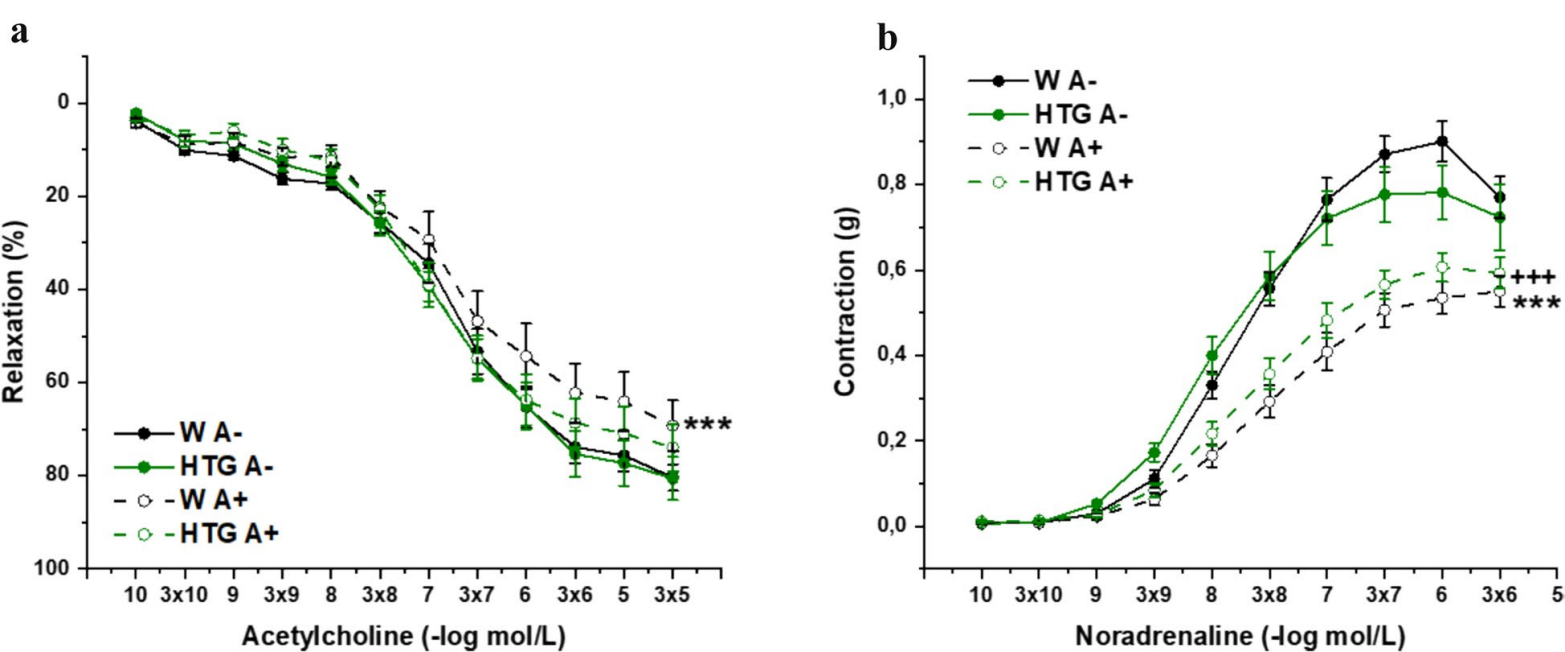
The role of dyslipidemia and PVAT in endothelium-dependent vasorelaxation (**a**) and adrenergic contraction (**b**) of isolated thoracic aorta in female Wistar and HTG rats. *W* Wistar female rats, *HTG* hereditary hypertriglyceridemic female rats, *A–* perivacular adipose tissue denuded rings, *A +* perivascular adipose tissue preserved rings. The results are presented as the mean ±S.E.M., and differences between groups were analyzed by three-way ANOVA. ***p < 0.001 vs W A–, ^+++^p < 0.001 vs HTG A–

In the next step, we monitored the influence of dyslipidemia and PVAT on vascular function in female rats. An evaluation of endothelial function revealed that the application of Ach (10^−10^–10^−5^ mol/L) relaxed the NA-precontracted TA rings in Wistar and HTG females differently. In HTG females, no difference in the relaxation response depending on the presence of PVAT was observed; however, in control females, endothelium-dependent relaxation was significantly reduced in rings with preserved PVAT (p< 0.001), and 3-way ANOVA revealed a significant effect of PVAT (F_(1,753)_ = 17.01; *p* = 4.15×10^–5^) but not dyslipidemia on endothelial function (F_(1,753)_ = 2.15; *p* = 0.14) (Fig. 3a). Similarly, we confirmed the significant effect of PVAT (F_(1,618)_ = 184.83; *p* → 0) but not dyslipidemia (F_(1,619)_ = 1.58; *p* = 0.21) on aortic contractility, and the Bonfferoni post hoc test revealed that, compared with rings without PVAT, rings with preserved PVAT had a significantly smaller response in both control (p< 0.001) and hypertriglyceridemic (p< 0.001) females (Fig. 3b).

### The role of NO signaling in the vascular function of female Wistar and HTG rats

**Fig. 4:**
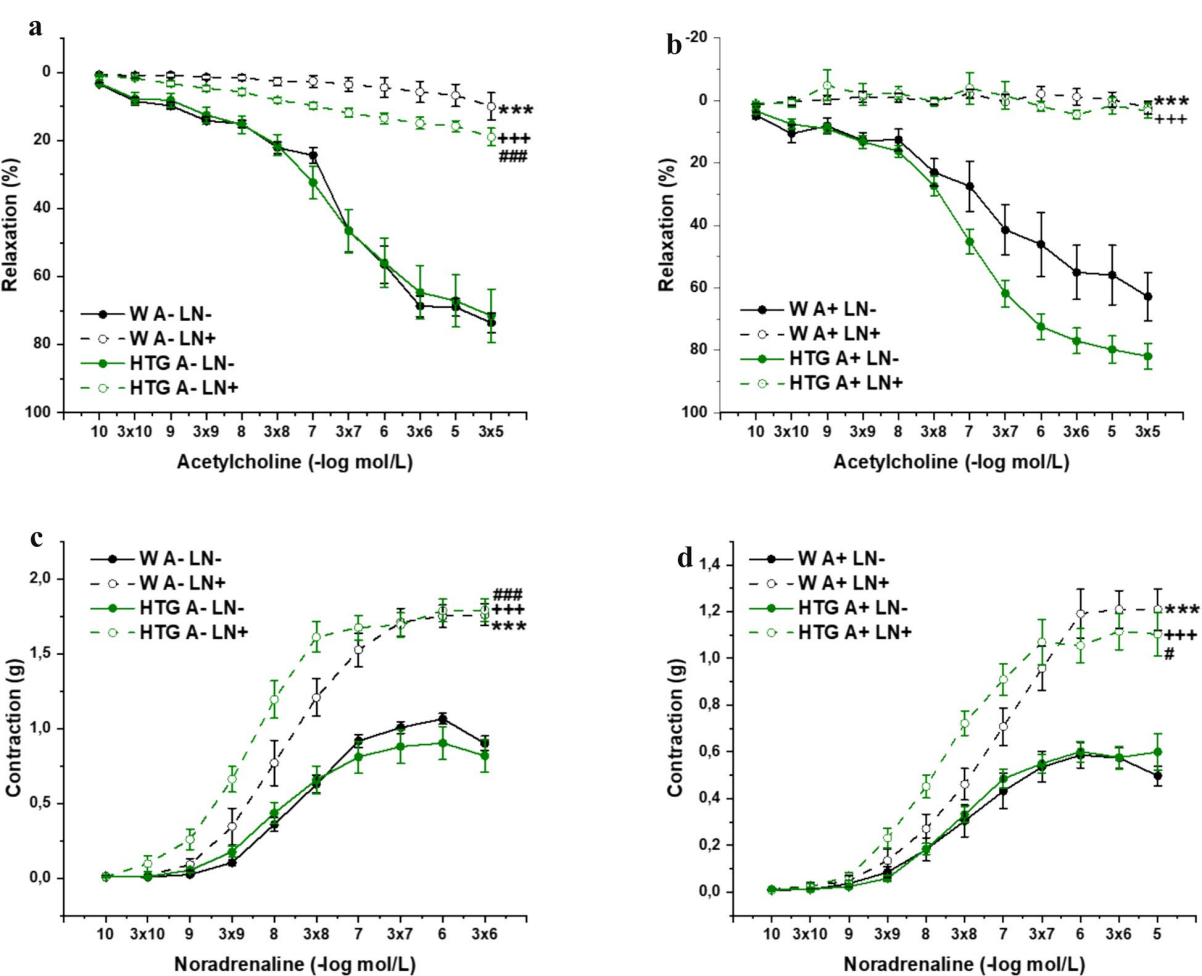
The role of NO signaling in endothelium-dependent vasorelaxation (**a**, **b**) and adrenergic contraction (**c**, **d**) of isolated thoracic aorta in female Wistar and HTG rats. *W* Wistar female rats, *HTG* hereditary hypertriglyceridemic female rats, *A-* perivascular adipose tissue denuded rings, *A +* perivascular adipose tissue preserved rings, *LN–* rings without incubation with NOS inhibitor (N^G^-nitro-L-arginin methyl ester), *LN+* rings incubated with NOS inhibitor (N^G^- nitro-L-arginin methyl ester). The results are presented as the mean ± S.E.M., and differences between groups were analyzed by three-way ANOVA. a, c: ***p < 0.001 vs W A– LN–, ^+++^p < 0.001 vs HTG A– LN–, ^###^p < 0.001 vs W A– LN + ; b, d: ***p < 0.001 vs W A + LN–, ^+++^p < 0.001 vs HTG A + LN–, ^#^p < 0.05 vs W A + LN +

The arterial rings were incubated with N^G^-nitro-L-arginine methyl ester (LN; 10^−4^ mol/L) for 20 minutes for analysis of NO signaling participation in vasoactive responses. In PVAT-denuded rings, three-way ANOVA confirmed a significant effect of NO inhibition (F_(1,382)_ = 908.11; p → 0) but not

dyslipidemia (F_(1,382)_ = 1.79; p = 0.18) on endothelial function. The Bonfferoni posthoc test revealed that treatment with the inhibitor significantly inhibited endothelium-dependent relaxation in both strains (both p< 0.001); however, in hypertriglyceridemic aortas significantly less (p< 0.001) (Fig. 4a). In the PVAT-preserved rings, three-way ANOVA confirmed a significant effect of both NO inhibition (F_(1,382)_ = 933.82; p → 0) and dyslipidemia (F_(1,382)_ = 23.62; p = 1.80×10^–6^) on endothelial function. The Bonfferoni posthoc test revealed that treatment with the inhibitor significantly inhibited endothelium-dependent relaxation similarly in both strains (both p< 0.001) (Fig. 4b). With respect to contractility in the PVAT- denuded rings, three-way ANOVA confirmed a significant effect of both NO inhibition (F_(1,305)_ = 454.45; p → 0) and dyslipidemia (F_(1,305)_ = 7.66; p = 0.006) on NA-induced contraction. Similarly, in the PVAT- preserved rings, three-way ANOVA confirmed a significant effect of both NO inhibition (F_(1,315)_ = 241.12; p → 0) and dyslipidemia (F_(1,315)_ = 4.77; p = 0.03). The Bonfferoni posthoc test revealed that, regardless of the presence of PVAT, treatment with the inhibitor significantly increased the contraction of both strains (both p<0.001); however, in hypertriglyceridemic aortas, the sensitivity to noradrenaline increased, and the curve was shifted to the left (PVAT-denuded rings: p<0.001; Fig. 4c; PVAT-preserved rings: p< 0.05; Fig. 4d).

**Fig. 5:**
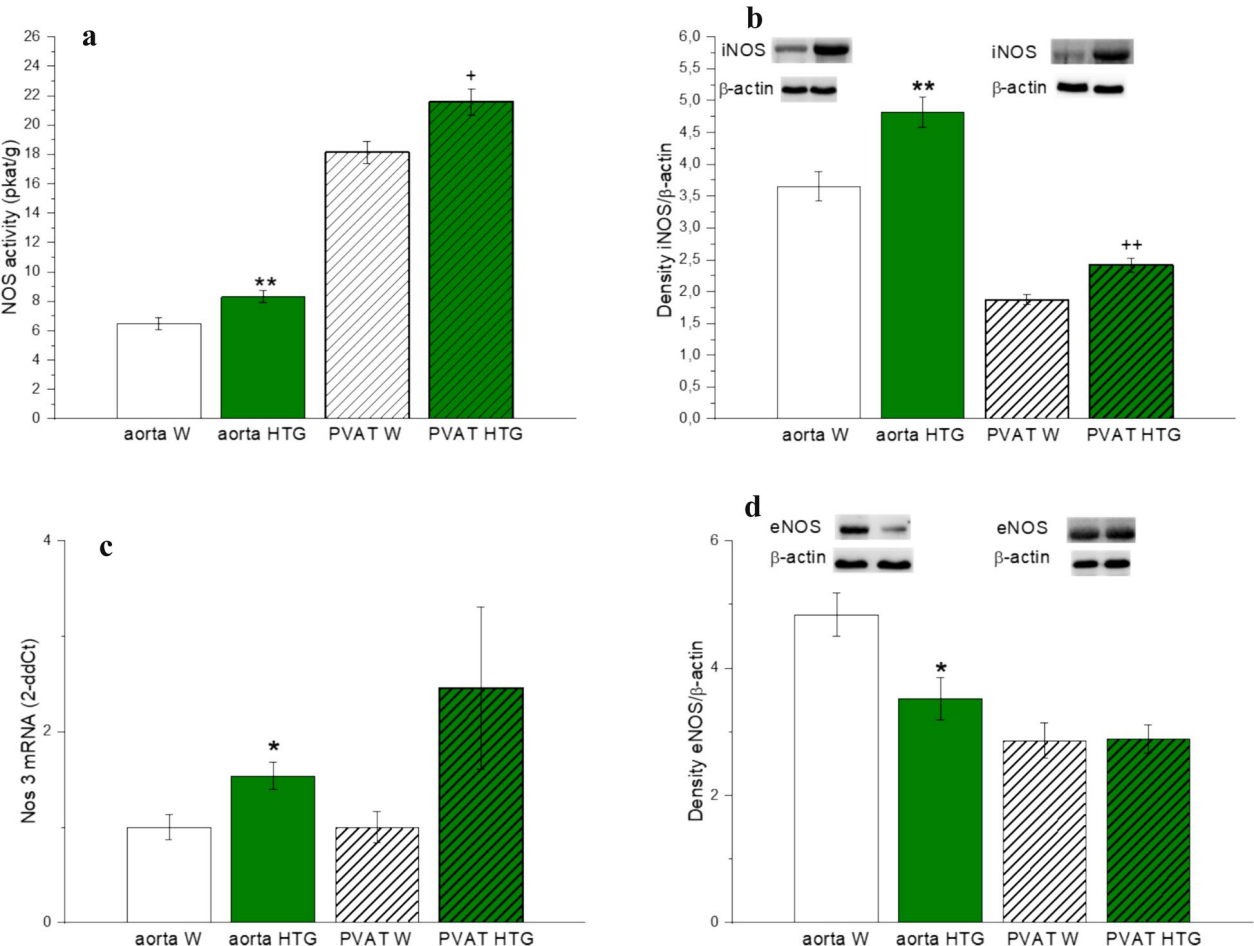
NO synthase activity (**a**), protein (**b**, **d**) and gene (**c**) expression of NO-synthase isoforms in aorta and PVAT in female Wistar and HTG rats. *W* Wistar female rats, *HTG* hereditary hypertriglyceridemic female rats, *PVAT* perivascular adipose tissue, *NOS* NO-synthase, *iNOS* inducible NO-synthase, *eNOS* endothelial NO-synthase, *Nos3 mRNA *the mRNA level of endothelial NO-synthase. The results are presented as the mean ±S.E.M., and differences between groups were analyzed by one-way ANOVA. *p < 0.05 vs aorta W, **p < 0.01 vs aorta W, ^+^p < 0.05 vs PVAT W, ^++^p < 0.01 vs PVAT W

The evaluation of total NO synthase activity revealed that it was significantly greater in HTG females in both aortic vascular tissue (p<0.01) and PVAT (p<0.05) (Fig. 5a), which was consistent with increased protein expression of the inducible NO synthase isoform in both tissues (both p<0.01) (Fig. 5b). On the other hand, although the mRNA level of the *Nos3* gene was increased in the aortic tissue of HTG rats (Fig. 5c), the protein expression of the endothelial isoform NO-synthase was decreased (Fig. 5d). In PVAT, both the gene and protein expression of the endothelial NO-synthase isoform were comparable between control and hypertriglyceridemic rats (Fig. 5c, d).

### The role of H_2_S signaling in the vascular function of female Wistar and HTG rats

**Fig. 6:**
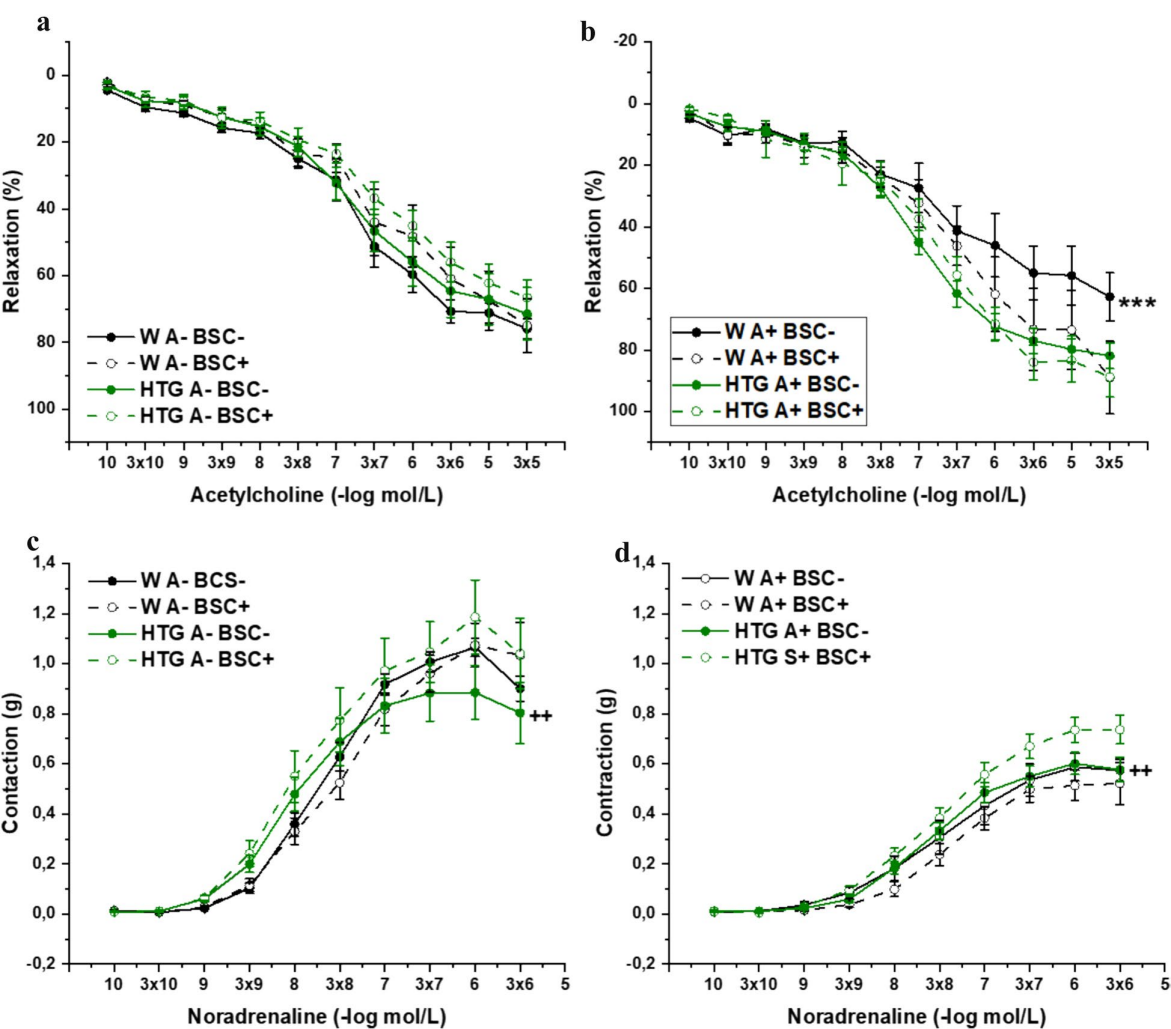
The role of H_2_S signaling in endothelium-dependent vasorelaxation (**a**, **b**) and adrenergic contraction (**c**, **d**) of isolated thoracic aorta in female Wistar and HTG rats. *W* Wistar female rats, *HTG* hereditary hypertriglyceridemic female rats, *A–* perivascular adipose tissue denuded rings, *A +* perivascular adipose tissue preserved rings, *BSC–* rings without incubation with H_2_S scavenger (bismuth(III) subsalicylate), *BSC+* rings incubated with H_2_S scavenger (bismuth(III) subsalicylate). The results are presented as the mean ± S.E.M., and differences between groups were analyzed by three-way ANOVA. ***p < 0.001 vs W A + BSC + , **c**: ^++^p < 0.01 vs HTG A- BSC + , **d**: ^++^p < 0.01 vs HTG A + BSC +

The arterial rings were incubated with H_2_S scavenger bismuth(III) subsalicylate (BSC, 10^−6^ mol/L) for 20 minutes to analyze H_2_S signaling participation in vasoactive responses. Although three-way ANOVA confirmed a significant effect of both BSC (F_(1,382)_ = 10.16; p = 0.002) and dyslipidemia (F_(1,382)_ = 4.82; p = 0.03) on endothelial function in the PVAT-denuded rings, the Bonfferoni posthoc test did not reveal significant differences between the groups (Fig. 6a). In the PVAT-preserved rings, three-way ANOVA also confirmed a significant effect of both BSC (F_(1,380)_ = 5.08; p = 0.02) and dyslipidemia (F_(1,380)_ = 18.23; p = 2.55×10^–5^) on endothelial function. The Bonfferoni posthoc test revealed that treatment with the inhibitor significantly inhibited endothelium-dependent relaxation in Wistar rats only (p<0.001) (Fig. 6b). With respect to contractility in the PVAT-denuded rings, three-way ANOVA confirmed a significant effect of BSC (F_(1,313)_ = 3.93; p=0.048) but not dyslipidemia (F_(1,314)_ = 3.08; p = 0.08) on noradrenaline-induced contraction. On the other hand, in the PVAT-preserved rings, three-way ANOVA confirmed a significant effect of dyslipidemia (F_(1,317)_ = 23.03; p = 2.63×10^–6^) but not BSC (F_(1,317)_ = 0.54; p=0.46). The Bonfferoni posthoc test revealed that treatment with the inhibitor significantly increased the contraction of hypertriglyceridemic rats regardless of the presence of PVAT (both p<0.01; Fig. 6c, d).

**Fig. 7:**
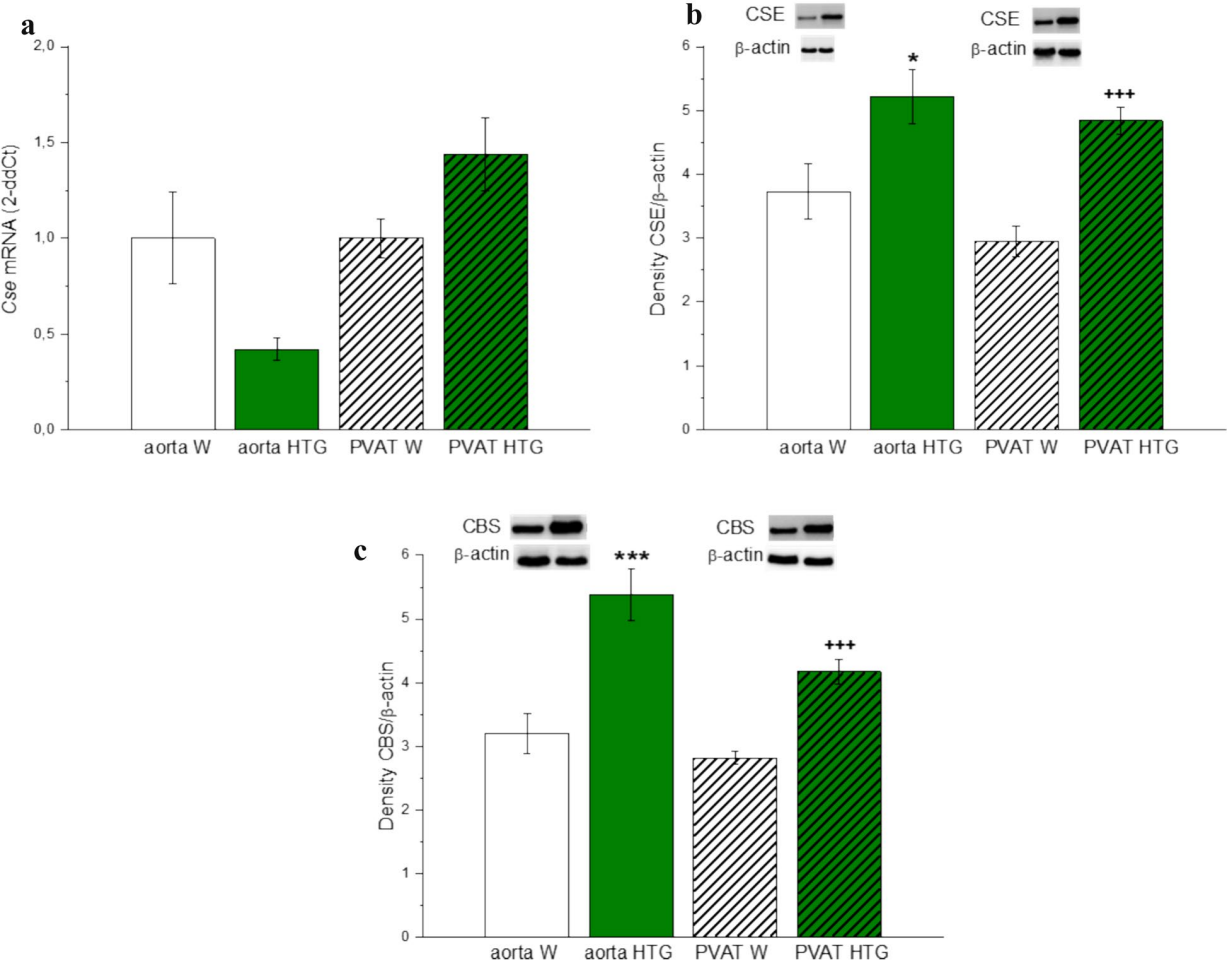
Gene (**a**) and protein (**b**, **c**) expressions of H_2_S producing enzymes in female Wistar and HTG rats. *W* Wistar female rats, *HTG* hereditary hypertriglyceridemic female rats, *PVAT* perivascular adipose tissue, *CSE* cystathionin-γ-lyase, *CBS* cystathionin-β-synthase, *Cse mRNA* the mRNA level of cystathionin-γ-lyase. The results are presented as the mean ± S.E.M., and differences between groups were analyzed by one-way ANOVA. *p < 0.05 vs aorta W, ***p < 0.001 vs aorta W, ^+^p < 0.05 vs PVAT W, ^+++^p < 0.001 vs PVAT W

Regarding the expression of H_2_S-producing enzymes in aortic tissue and PVAT, our results confirmed that although the mRNA levels of the *Cse* gene revealed a borderline decrease in aortic tissue (p=0.0542) or no change in PVAT in HTG rats compared with those in Wistar rats (Fig. 7a), the protein expression of CSE was significantly increased in both aortic tissue (p<0.05) and PVAT (p<0.001) in HTG rats (Fig. 7b). Similarly, the protein expression of CBS was increased in both the aortic tissue (p<0.001) and PVAT (p<0.001) of HTG rats (Fig. 7c).

### The role of COX signaling and inflammation in the vascular function of female Wistar and HTG rats

**Fig. 8:**
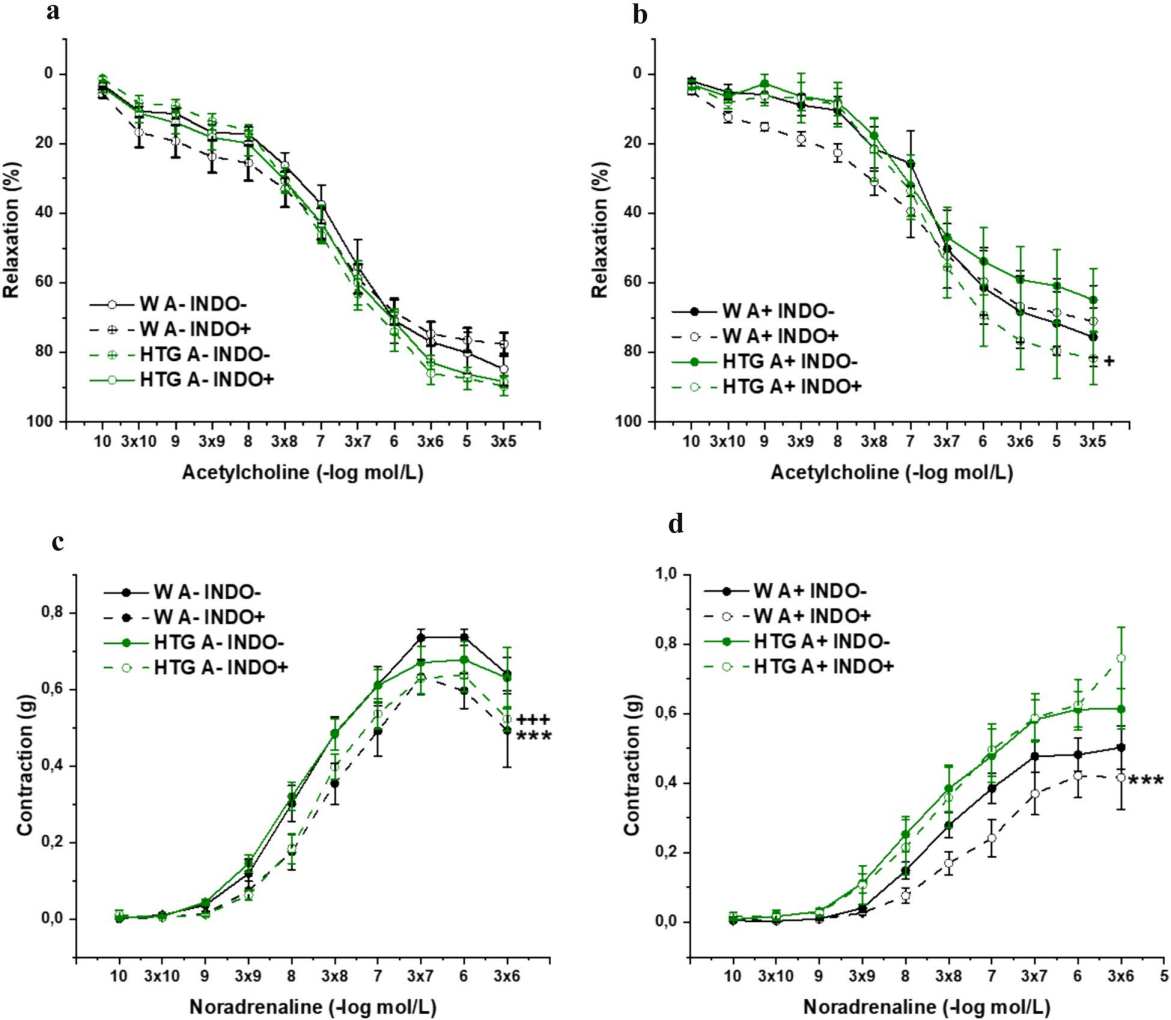
The role of COX signaling in endothelium-dependent vasorelaxation (**a**, **b**) and adrenergic contraction (**c**, **d**) of isolated thoracic aorta in female Wistar and HTG rats. *W* Wistar female rats, *HTG* hereditary hypertriglyceridemic female rats, *A–* perivascular adipose tissue denuded rings, *A+* perivascular adipose tissue preserved rings, *INDO–* rings without incubation with COX inhibitor (indomethacin), *INDO+* rings incubated with COX inhibitor (indomethacin). The results are presented as the mean ± S.E.M., and differences between groups were analyzed by three-way ANOVA. **c**: ***p < 0.001 vs W A– INDO–, ^+++^p < 0.001 vs HTG A– INDO–, **b**, **d**: ***p < 0.001 vs W A+ INDO–, ^+^p < 0.05 vs HTG A+ INDO–

The arterial rings were incubated with the COX inhibitor indomethacin (INDO, 10^−5^ mol/L) for 20 minutes to analyze the participation of cyclooxygenase signaling in vasoactive responses. In the PVAT-denuded rings, three-way ANOVA did not confirm a significant effect of INDO (F_(1,382)_ = 1.87; p = 0.17) or dyslipidemia (F_(1,2.55)_ = 2.55; p = 0.11) on endothelial function, and the Bonfferoni posthoc test also revealed no significant differences between the groups (Fig. 8a). On the other hand, in the PVAT- preserved rings, 3-way ANOVA confirmed a significant effect of INDO (F_(1,334)_ = 8.21; p = 0.0045) but not dyslipidemia (F_(1,334)_ = 1.59; p = 0.21) on endothelial function. The Bonfferoni posthoc test revealed that treatment with the inhibitor significantly increased endothelium-dependent relaxation in HTG rats, indicating the anti-relaxant properties of the COX products (p<0.05) (Fig. 8b). With respect to contractility in the PVAT-denuded rings, 3-way ANOVA confirmed a significant effect of INDO (F_(1,310)_ = 39.80; p=1.14×10^–9^) but not dyslipidemia (F_(1,310)_ = 0.11; p = 0.74) on noradrenaline-induced contraction. On the other hand, in the PVAT-preserved rings, 3-way ANOVA confirmed a significant effect of dyslipidemia (F_(1,287)_ = 54.11; p = 2.74×10^–12^) but not INDO (F_(1,287_ = 3.84; p=0.051). The Bonfferoni posthoc test revealed that treatment with the inhibitor significantly decreased contraction in Wistar rats regardless of the presence of PVAT (both p<0.001; Fig. 8c, d). On the other hand, whereas treatment with the inhibitor significantly decreased contraction in the PVAT-denuded rings of HTG rats (p<0.001) so demontsrating pro-contractile action of COX products, the presence of PVAT eliminated this effect (Fig. 8c, d). In addition, our results revealed that the protein expression of COX_2_ was significantly increased in both the aortic (p<0.001) and PVAT (p<0.05) tissues of HTG rats (Table 2).

**Table 2:** Gene and protein expressions of inflammatory factors in female Wistar and HTG rats

Parameters	Aorta W	Aorta HTG	PVAT W	PVAT HTG
COX_2_ (Density COX2/β-actin)	6.30 ± 0.45	9.77 ± 0.37***	0.59 ± 0.03	1.01 ± 0.09^+^
ICAM (Density ICAM/β-actin)	2.39 ± 0.26	4.35 ± 0.26***	5.99 ± 0.12	5.44 ± 0.16^+^
*Mcp-1*mRNA (2-ddCt)	1.00 ± 0.15	0.69 ± 0.29	1.00 ± 0.31	1.74 ± 0.31
Mcp-1 (Density Mcp-1/β-actin)	0.77 ± 0.13	1.86 ± 0.21***	0.66 ± 0.05	0.50 ± 0.03^+^
*TNFα*mRNA (2-ddCt)	1.00 ± 0.24	0.27 ± 0.03*	1.00 ± 0.37	0.60 ± 0.15
TNFα (Density TNFα/β-actin)	0.98 ± 0.10	3.50 ± 0.17***	1.03 ± 0.15	6.08 ± 0.26^+++^
*NFκB*mRNA (2-ddCt)	1.00 ± 0.17	1.40 ± 0.28	1.00 ± 0.15	1.17 ± 0.18
NFκB (Density NFκB/β-actin)	6.31 ± 0.17	10.48 ± 0.23***	3.40 ± 0.26	7.96 ± 0.58^+++^

*COX*_*2*_ cyclooxygenase 2, *ICAM* intercellular adhesion molecule, *Mcp-1* monocyte chemoattractant protein-1, *TNFα* tumor necrosis factor alpha, *NfκB* nuclear factor kappa B, *W* Wistar female rats, *HTG* hereditary hypertriglyceridemic female rats, *PVAT* perivascular adipose tissue The results are presented as the mean ±S.E.M., and differences between groups were analyzed by one-way ANOVA. *p < 0.05 vs aorta W, ***p < 0.001 vs aorta W, ^+^p < 0.05 vs PVAT W, ^+++^p < 0.001 vs PVAT W

We also evaluated the gene and protein expression of several inflammatory factors. The relative gene expression of *Tnfα* was decreased in the aorta tissue (p<0.05) of HTG female rats and did not differ in the PVAT tissue between Wistar and HTG females. The relative mRNA expression of *Mcp-1* and *NfκB* was comparable in both the aorta and PVAT tissues of Wistar and HTG female rats. Although increased protein expression of TNFα and NfκB was confirmed in the aortic and PVAT tissues, the protein expression of factors regulating the adhesion and infiltration of monocytes (ICAM-1 and MCP-1) was decreased in the PVAT tissue (Table 2).

## Discussion

### Sex differences in prediabetic rats

In our study, HTG rats of both strains were characterized by dyslipidemia, impaired glucose tolerance, and hypertension. However, while changes in the lipid profile (increased TAG levels and decreased HDL-C levels) were not affected by sex, HTG females presented milder hypertension and better glucose utilization than males did. Komnenov and Rossi [14] demonstrated that, compared with male rats, adult female Sprague‒Dawley rats were protected from the effects of fructose and a high-salt diet on BP, aortic stiffness, and ventricular remodeling. Similar protection with respect to cardiac remodeling was observed in HTG females in this study. We previously reported hypotrophy of the myocardium in HTG males, which was associated with an increased plasma level of alanine aminotransferase, a marker of damage to, among other tissues, the myocardium [15]. The literature shows that hypertriglyceridemia is associated with increased accumulation of lipids in nonfat tissues such as the liver, heart, muscles and kidneys, which leads to lipotoxicity and tissue damage [16, 17]. However, on the basis of our findings, prediabetic females may develop protective mechanisms to reduce the negative impact of dyslipidemia on the cardiovascular system, which is likely related to the fact that estrogens can partially prevent an increase in BP [18].

With respect to vascular function, our results confirmed that females, regardless of strain, had better preserved endothelial function than males in which endothelial dysfunction was noted. Moreover, the presence of PVAT inhibited endothelium-dependent relaxation in all the rats except HTG females. With respect to the effect of PVAT, several studies have shown that in impaired metabolic conditions, pathological processes contributing to impaired vascular function are activated in PVAT. Ketonen et al. [19] reported that, in the presence of PVAT, the aortas of obese male mice revealed impaired endothelium-dependent vasodilation that was restored after the removal of PVAT and after the reduction of superoxide and hydrogen peroxide formation. A study by DeVallance et al. [20] demonstrated that aortic rings from male obese Zucker rats incubated with their PVAT-conditioned media revealed endothelial dysfunction, which was associated with aortic stiffness and altered inflammatory signaling in PVAT. On the other hand, similar to our results, Tilley et al. [21] demonstrated a lack of PVAT-mediated vascular dysfunction in obese Zucker female rats, which was accompanied by an altered PVAT phenotype from brown to white. Generally, hypertriglyceridemia in male rats is associated with impaired endothelial function associated with marked changes in vascular architecture and PVAT-associated oxidative stress [22, 23]. On the other hand, in female prediabetic rats, dyslipidemia does not lead to the development of endothelial dysfunction, which is partly related to the presence of PVAT.

In addition to endothelial function, the contractility results also confirmed the presence of protective mechanisms in female HTG rats. Conflicting data have generally been reported regarding sex differences in responses to vasoconstrictor factors, and investigators reported both an increase [24] and a decrease [25] in aortic responses to vasoconstrictors in male rats compared with females. In our study, pathologically increased aortic contractility was noted in HTG males compared with Wistar males similar to our previous studies where the augmented vasoconstriction was associated with arterial wall thickening [26]. Moreover, high levels of triacylglycerols can modulate the cytosolic free calcium concentration ([Ca^+^]i) by affecting various aspects of calcium handling, including increased calcium influx and store mobilization which is associated with increased contraction [27]. On the other hand, despite the development of dyslipidemia, we surprisingly demonstrated significant anticontractile activity of PVAT in HTG rats regardless of sex. Our observations contrast with data reported in the literature, which mostly revealed a reduced anticontractile effect of PVAT in experimental models of metabolic disorders or diabetes [3, 28]. However, the nonobese HTG rats used in this study represent an early stage of metabolic syndrome and prediabetes in which metabolic changes have not reached a high degree of disorder and can develop compensatory vasoactive mechanisms. Similar results were confirmed in SHRs, in which the PVAT of mesenteric arteries revealed stronger anticontractile activity to counteract increased vascular tone [4]. In this study, the most developed compensatory mechanisms were observed in female HTG rats, where contractile responses approached the physiological values of control rats. Similar beneficial effects in females were observed by Watts et al. [29] in Dahl SS rats fed a high-fat diet, where they were associated with PVAT activity. The authors demonstrated anticontractile activity in both sexes; however, the contractions of thoracic aortas were smaller in females than in males. Moreover, the beneficial function of PVAT in promoting stress relaxation was preserved only in females, which was probably related to the change in the adipocyte phenotype (again from brown to white). Taken together, our study revealed that the protective vasoactive mechanisms manifested by improved endothelial function and contractility are triggered in female HTG rats. Although multiple signaling pathways may be involved in these mechanisms, in our study, we focused on the potential roles of NO, H_2_S, COX and inflammatory signaling.

### The role of NO, H_2_S, inflammation and COX signaling in the vascular function of prediabetic females

In our previous study, we confirmed that NO synthase activity in both the arterial wall and PVAT tissue was significantly inhibited in male HTG rats, which likely worsened endothelial relaxation [5]. Estrogens are known to have a beneficial effect on vascular endothelial function in females and can increase NO synthase (NOS) activity in arterial tissue [30] as confirmed by our findings in in both aortic and PVAT tissues. It was associated with increased sensitivity of HTG aortas to noradrenaline during acute NO inhibition, which is consistent with the findings of Sudhir et al. [31], who reported that estrogens can increase basal NO release. We also confirmed preserved endothelial function, to which in the PVAT- denuded rings NO contributed to a lesser extent than in controls. On the other hand, the presence of PVAT eliminated this difference, which was consistent with the finding that the expression of the endothelial NOS isoform was reduced in aortic tissue but not in PVAT. Similar PVAT-dependent compensation for endothelial dysfunction was reported by Kagota et al. [32] in the mesenteric artery of female SHRSP.ZF rats, a metabolic syndrome model in which the presence of PVAT enhances relaxation in females but not in males. The authors also confirmed that one of the predictors of beneficial effects was the expression of apelin in PVAT, which, as shown by Tatemoto et al. [33], can stimulate vasorelaxation via increased NO production. However, in our study, the increased NOS activity could also be due to increased protein expression of iNOS. In the aortic wall and PVAT of HTG females we confirmed, despite the unchanged or decreased gene expression, the increased protein expression of TNF-α and NfκB, proinflammatory cytokines which are the main inducers of iNOS expression [34]. In the HTG aortic wall, we also observed significantly increased protein expression of other inflammatory factors, ICAM-1 and MCP-1, which regulate the adhesion and infiltration of monocytes/macrophages. In contrast, the protein expression of both factors was decreased in PVAT. Similar results were reported by Vieira-Potter et al. [35], who confirmed that high-fat diet feeding of female Ossabaw pigs caused a significant upregulation of genes associated with inflammation in arterial tissue, however, these genes were not elevated, and many were reduced in PVAT. All above mentioned findings point to the importance of PVAT in the (non)functionality of inflammatory factors within the arterial tree as well as in mediating the protective effects of NO.

In pathological conditions, impaired vascular function may also be associated with the prevalence of vasoconstrictors produced by cyclooxygenase (COX). Increased levels of COX-derived products such as PGH_2_ and TXA_2_, which contribute to increased vascular contractility [36] and impairment of endothelium-dependent relaxation [37], are found in experimental diabetes. Moreover, our previous results, as well as those of other authors, confirmed the proinflammatory role of COX2 [38, 39]. In our study, we revealed the participation of pro-contractile COX products in HTG aortas, where they generally contributed via pro-contractile/anti-relaxant actions, which was accompanied by increased protein expression of COX_2_ in both aortic and PVAT tissues. In line with our results, Gallo et al. [40] demonstrated that COX_2_ was overexpressed in the aorta of diabetic rats, which was associated with altered Na^+^/K^+^-ATPase activity, an enzyme that contributes to the regulation of vascular contractility. However, despite the pro-contractile action of COX products confirmed in this study, as well as by other authors, the vasoactive responses of female HTG rats approached physiological values, thus confirming the presence of additional balancing mechanisms.

In addition to NO, another pathway that could play a significant role in compensating for the metabolic changes associated with hypertriglyceridemia is sulfide signaling. Our previous studies revealed that H_2_S signaling can be stimulated in different pathological conditions, e.g., in fructose-fed normotensive rats [41] as well as in SHRs, where the increased participation of H_2_S in vasorelaxation could counterbalance endogenous NO deficiency [42] or contribute to maintaining aortic endothelial function during ACE_2_ inhibition [43]. With respect to HTG males, we previously confirmed a dual effect depending on the type of triggered signaling pathway: H_2_S within the arterial wall contributes to endothelial dysfunction; however, H_2_S released from PVAT has been shown to have anticontractile effects to compensate for pathologically increased contractility of the thoracic aorta [5]. In this study, we confirmed that, regardless of the presence of PVAT, endogenous H_2_S had beneficial anticontractile effects, which were associated with increased protein expression of H_2_S-producing enzymes in both aortic and PVAT tissues. Moreover, unlike in the Wistar rats where H_2_S produced by PVAT reduced endothelium-dependent relaxation, in HTG rats H_2_S produced by PVAT did not inhibit endothelial function. We assume that a lower level of H_2_S in the PVAT and aortic tissue of Wistar rats could affect NO action. Szijártó et al. [44] demonstrated that one of the major functions of H_2_S produced by CSE is to reduce endothelial NO bioavailability by the direct interaction of H_2_S and NO while the inhibitory effect of H_2_S on NO signaling is mainly manifested at its low concentrations [45]. On the other hand, the higher level of H_2_S in HTG aortas could change the regulatory effect of H_2_S which led to the elimination of anti-relaxation effect and manifestation of a beneficial anticontractile action. Taken together, it seems that the beneficial effect of endogenously produced H_2_S was stronger in females than in males. This finding may be partially related to a higher level and poorer utilization of glucose in males than in females. Emilova et al. [28] confirmed that the anticontractile effect of H_2_S became pro-contractile due to the hyperglycemic environment of streptozotocin-induced diabetic rats. Nevertheless, as mentioned above, estrogen plays a key role in cardiovascular protection, and Li et al. [46] reported that there is a link between the beneficial effects of estrogens and the activity of the sulfide signaling pathway. The authors confirmed that the anti-atherosclerotic effect of estrogen was mediated by CSE-generated H_2_S and that estrogen treatment of atherogenic diet-fed mice increased H_2_S production in vascular tissues. Moreover, the authors reported that the inhibitory effect of estrogen on inflammation, oxidative stress and dyslipidemia correction required a functionally intact CSE/H_2_S system. Another study similarly confirmed that treatment with 17β-estradiol enhanced acetylcholine vasorelaxation of the femoral artery in ovariectomized mice by stimulating endothelial H_2_S release, which was associated with PKG activation [47]. Finally, deOliveira et al. [48] reported that endothelial dysfunction in hypertensive pregnant rats was compensated by PVAT-dependent stimulation of endogenous H_2_S signaling, Taken together, these studies confirm the importance of the H_2_S system in the protective regulation of vascular tone in females. The results of our work provide new information that the activation of the sulfide signaling pathway represents an important compensatory vasoactive mechanism against hypertriglyceridemia-associated metabolic disorders.

## Conclusion

Our study confirmed that despite increased inflammation and the negative impact of the cyclooxygenase pathway, HTG female rats trigger protective vasoactive mechanisms associated with mild hypertension, cardiac protection and improved vascular function. The improved endothelial function and contractility were associated with PVAT activity, where suppressed expression of specific inflammatory factors has been demonstrated. Finally, the sulfide and nitroso signaling pathways represent important compensatory vasoactive mechanisms that successfully balance hypertriglyceridemia-associated metabolic disorders and may be promising therapeutic targets in prediabetic females.

## Data Availability

The data used to support the findings of this study are available from the corresponding author upon request.

## Abbreviations

A–: Perivascular adipose tissue removed rings
A+: Perivascular adipose tissue preserved rings
Ach: Acetylcholine
BP: Blood pressure
BSC: Bismuth(III) subsalicylate
BW: Body weight
CBS: Cystathionin-β-synthase
Chol: Total cholesterol
COX: Cyclooxygenase
CSE: Cystathionin-γ-lyase
H_2_S: Hydrogen sulfide
HDL-C: High-density lipoprotein cholesterol
HTG: Hereditary Hypertriglyceridemic rats
HW: Heart weight
INDO: Indomethacin
L-NAME: N^G^-nitro-L-arginine-methyl ester
NA: Noradrenaline
NO: Nitric oxide
NOS: Nitric oxide synthase
OGTT AUC: Oral glucose tolerance test-area under curve
PVAT: Perivascular adipose tissue
SBP: Systolic blood pressure
TA: Thoracic aorta
TAG: Triacylglycerols
